# Cyclo­hexane plastic phase I: single-crystal diffraction images and new structural model

**DOI:** 10.1107/S2414314623001141

**Published:** 2023-03-02

**Authors:** Sylvain Bernès, Sebastian Camargo

**Affiliations:** a Instituto de Física Luis Rivera Terrazas, Benemérita Universidad Autónoma de Puebla, 18 Sur y San Claudio S/N, Puebla, Pue. 72570, Mexico; Utrecht University

**Keywords:** cyclohexane, plastic crystals, X-ray diffraction, diffraction image, disorder

## Abstract

The plastic phase of cyclohexane (polymorph I) was studied by Kahn and co-workers, without achieving a satisfactory determination of the atomic coord­inates [Kahn *et al.* (1973). *Acta Cryst*. B**29**, 131–138]. The positions of the C atoms cannot be determined directly as a consequence of the disorder in a high-symmetry space group, an inherent feature of plastic materials. Given this situation, the building of a polyhedron describing the disorder was the main tool for determining the molecular structure in the present work. Based on the shape of reflections {111}, {200} and {113} in space group *Fm* 3 *m*, we assumed that cyclohexane is disordered through the action of rotation group 432. The polyhedral cluster of disordered molecules is then a rhombic dodecahedron centred on the nodes of an fcc Bravais lattice. The vertices of this polyhedron are the positions of C atoms for the cyclohexane molecule, which is disordered over 24 positions. With such a model, the asymmetric unit is reduced to two C atoms placed on special positions, and an acceptable fit between the observed and calculated structure factors is obtained.

## Abstract 2

Diffraction images for the plastic phase I of cyclo­hexane at 255 K show that molecules are disordered over 24 positions, through four-, three- and twofold rotation axes in space group *Fm* 3 *m*, forming clusters in which the C atoms are located on the vertices of a rhombic dodecahedron.

## Graphical abstract

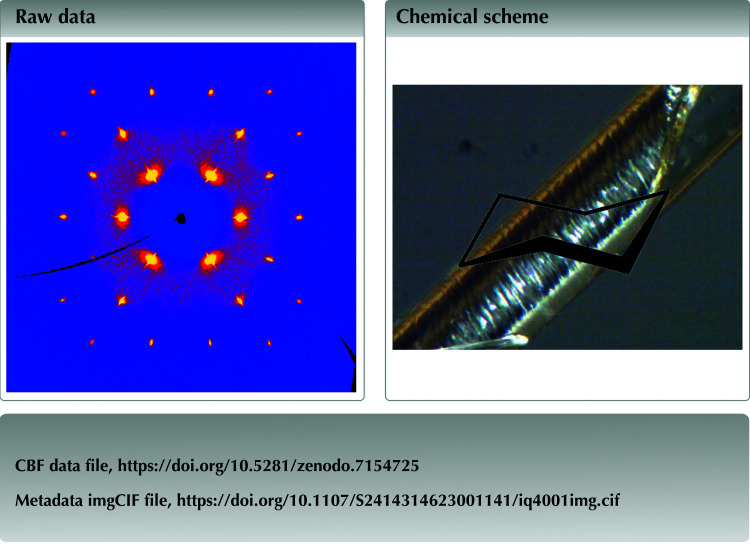

## Introduction

The concept of an organic ‘plastic crystal’ was first stated by J. Timmermans in 1938, although the name was coined ten years later by A. Michils, due to the mechanical softness of these materials. They share many physicochemical features with liquid crystals, and were indeed first described as a new mesomorphic state of matter. Thermodynamically, they are characterized by a very low entropy of fusion, Δ*S*_*m*_ < 5 eu (1 eu = 4.185 J mol^−1^ K^−1^), which was interpreted as the signature that a quasi-isotropic state, similar to that of liquids, is set just below their melting-point temperature (Timmermans, 1961 ▸). Mechanically, these materials behave as plastic metals and can be extruded at quite low pressures (Michils effect; Michils, 1948 ▸). Most often, the molecules concerned belong to high-symmetry point groups, and present a more or less globular shape while being orientationally disordered around their rotation axis; they have a marked propensity for polymorphism, with the form close to the melting point crystallizing in a high-symmetry space group, usually in the cubic crystal system, with a highly disordered crystal structure.

Cyclohexane, C_6_H_12_, is an emblematic example of such crystals. The ground state of the molecule is the rigid-chair conformer, belonging to 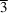 *m* (*D*_3*d*_) point group. The high-temperature phase I, in space group *Fm* 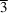 *m*, undergoes an isothermal transition at 186 K to the low-temperature ordered phase II, in space group *C*2/*c* (Kahn *et al.*, 1973 ▸). The entropy of fusion, Δ*S*_*m*_ = 2.29 eu, is much lower than that measured for the II 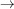 I transition, Δ*S*_II 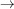 I_ = 8.66 eu (Ruehrwein & Huffman, 1943 ▸).

Early literature regarding the crystallographic characterization of cyclo­hexane phase I (Hassel & Sommerfeldt, 1938 ▸; Oda, 1948 ▸; Renaud & Fourme, 1966 ▸; Kahn *et al.*, 1973 ▸), systematically complains about technical hurdles related to the very nature of plastic crystals: (i) a very rapid fall-off of diffraction intensity with increasing Bragg angle; (ii) a scattering background blackening the photographic plates and masking weak reflections; (iii) the extreme difficulty of obtaining a reliable set of atomic coordinates, as a direct consequence of the previously mentioned issues. Indeed, only one article explicitly suggests a structural model based on atomic coordinates (Kahn *et al.*, 1973 ▸), which is discussed further below.

During our work on the structure prediction and crystallographic characterization of cyclo­alkanes that are liquids at room temperature (Camargo, 2018 ▸), we were able to obtain diffraction frames for the plastic phase I of cyclo­hexane. A careful examination of the reciprocal space rebuilt from these raw data offers greater insight into how molecules behave in the plastic phase, and allowed us to propose a new simple model explaining how molecules are disordered about the nodes of the fcc Bravais lattice.

## Crystallization and data collection

Anhydrous cyclo­hexane (reference 227048, Sigma-Aldrich, 99.5%) has a melting point close to 279 K. The end of a 0.4 mm diameter glass Lindemann capillary tube was filled with liquid cyclo­hexane and the head of the capillary sealed with wax, while avoiding any contamination of cyclo­hexane. The capillary was mounted on a standard goniometer head, and cyclo­hexane was crystallized *in situ*, on a Stoe Stadivari diffractometer equipped with an Oxford Cryosystems Cobra cooling device. No head spinning was applied during crystallization, and a key condition was to keep the capillary horizontally (χ = −90°), in order to have the N_2_ flow approximately normal to the capillary. In a first step, successive cycles of cooling/heating ramps with different rates were applied to obtain a powdered sample: from room temperature to 260 K at 360 K h^−1^, and then to 270 K at 200 K h^−1^. These microcrystals were then carefully merged by heating the sample to 273 K (60 K h^−1^) and then to 274 K (2 K h^−1^). Once a single crystal is stabilized in the capillary, the sample can be cooled to 260 K at 10 K h^−1^ and then to 250 K at 20 K h^−1^. We found that this methodology affords large and good-quality single crystals in a reproducible manner.

Diffraction intensities for one crystal were collected at 255 K with Ag *K*α radiation (AXO microfocus source equipped with multilayer ASTIX-f optics) and a PILATUS 100 K detector (487 × 195 pixels), accumulating 1139 frames over 19 h, each one being collected over 60 s, with a scan range of 1° in ω. Another crystal was collected at 245 K over 91 h. For this experiment, a very long exposure time of 1800 s per frame was used, with a scan range of 2° in ω. A set of 183 frames was collected for this crystal. Both data sets afford virtually the same structure refinement. The refinement reported in this paper is based on the first data set. The second data set is used herein for Fig. 3 only.

## Data processing

**Figure 1:**
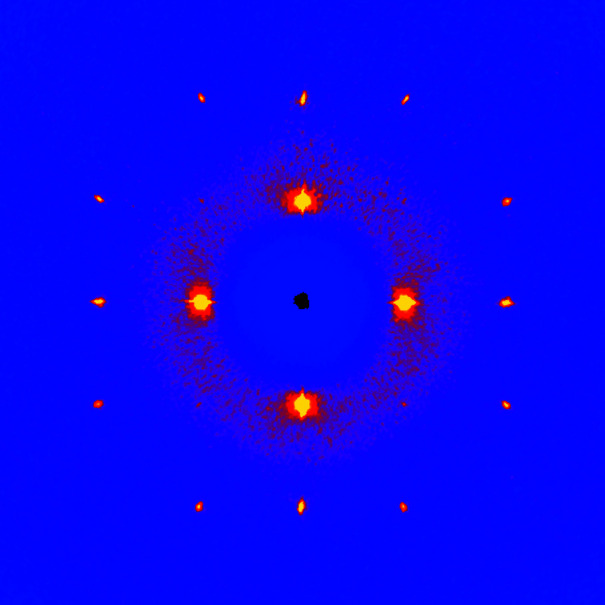
The (*hk*0) layer of reciprocal space for the plastic phase of cyclo­hexane, at 255 K. Direction [100] runs on the right, direction [010] runs upward.

Reciprocal space for each crystal was built using all collected frames, with the dedicated *X-AREA* tool (Stoe & Cie, 2019 ▸). A cubic 3D array centred on the origin of reciprocal space, with boundaries at −0.75 and +0.75 Å^−1^ (2θ_max_ = 42.7°) and a pixel resolution of 0.003 Å^−1^ was computed. Each detector pixel was divided into 10 subpixels in the plane of the detector, and into 20 subpixels in the direction normal to that plane. The resulting 3D arrays contain approximately 125 × 10^6^ voxels. Images in Figs. 1 ▸–3 ▸ ▸ are plotted using a conventional blue/yellow heat map.

Structure factors were obtained by integrating the 1139 frames collected on the first crystal. Elliptical integration masks are used, with the smallest diameter given by *W* = *A* + *B*tan θ and the largest diameter calculated as *W*/cos2θ + (Δλ/λ)tan θ, with *A* = 5 and *B* = −8. A rather large mosaic spread parameter was applied (ems = 0.048 rad), to take into account the plastic nature of the crystal. Finally, the background area was systematically limited to one pixel around the peak area. Intensities were scaled in the *m* 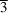 *m* Laue class in a standard way.

## Data description

**Figure 2:**
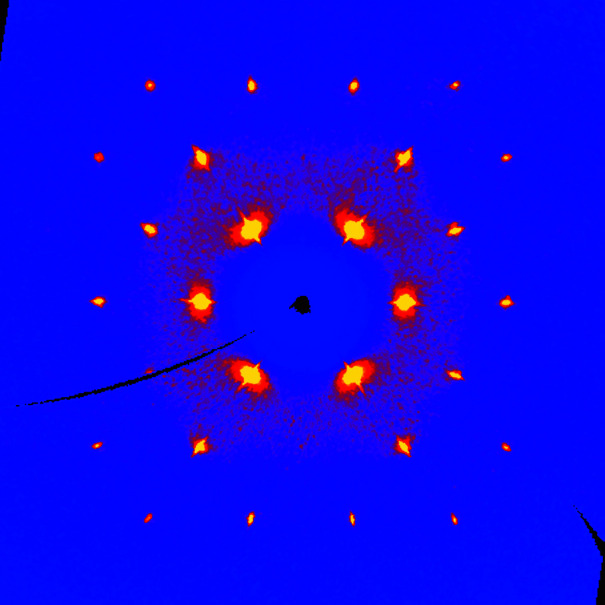
Reciprocal space of the plastic phase of cyclo­hexane, at 255 K, viewed in a projection normal to [011]. Direction [100] runs on the right, direction [01 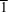 ] runs upward.

**Figure 3:**
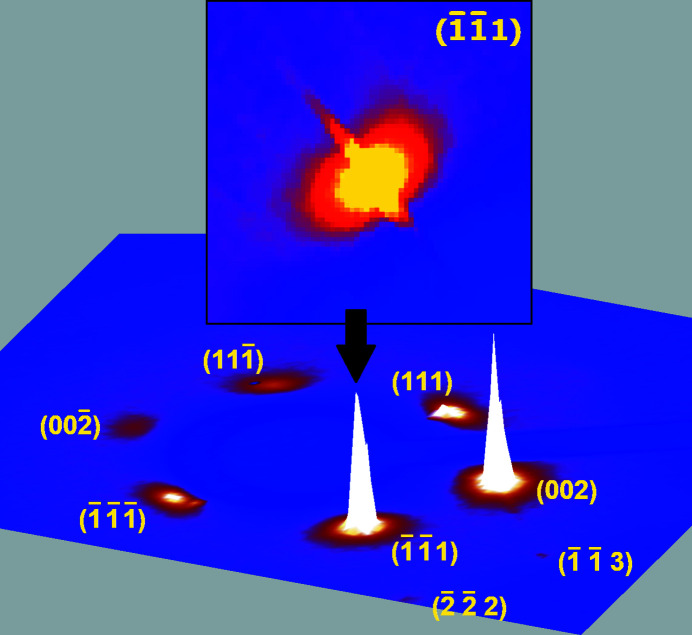
A single frame collected over 2° in ω over 1800 s for the plastic phase of cyclo­hexane at 245 K (bottom). Only the small part of the frame containing Bragg peaks is shown. The 3D plot of the frame (CBF format) was obtained using *CAP Frame View* v. 1.3 (Rigaku OD, 2015 ▸). Top inset: Reflection ( 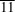 1) rebuilt using 183 collected frames. The projection is normal to [0 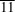 ], with [100] running on the right and [0 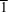 1] running upward. This image was generated using *X-AREA* (Stoe & Cie, 2019 ▸).

The (*hk*0) layer built with 1139 frames (Fig. 1 ▸) clearly shows that a single crystal was grown. Bragg peaks are well defined, although the resolution is, as expected, very low: the last observed reflections in the full pattern are (333) and (511), corresponding to a resolution of 1.67 Å. That resolution is not improved if frames are collected over 1800 s instead of 60 s. Moreover, this is exactly the same resolution as that obtained by Kahn *et al.* in 1973 ▸, and should thus be regarded as an intrinsic limit imposed by the plastic nature of the material. On the other hand, a homogeneous background is visible for 2θ < 12.5°, indicative of a degree of disorder, or, at the very least, indicative of large atomic motions in the crystal. Another projection of the reciprocal space (Fig. 2 ▸) shows an interesting feature: the reflections with highest intensities, {111}, display a rod-like diffuse scattering streak along 〈111〉, which should be related to the main direction for disorder (Welberry & Butler, 1995 ▸; Welberry & Goossens, 2014 ▸). These diffuse streaks are better visualized using data collected with long-exposure frames (Fig. 3 ▸). Assuming that disorder occurs exclusively through the crystallographic rotation axis, involved symmetry elements that are not parallel to 〈111〉 in space group *Fm* 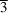 *m* are two- and fourfold axes. However, threefold axes are also used for disordering the molecule, as showed by the diffuse halo wrapping the {111} peaks. Although, to a lesser extent, {200} and {113} reflections also show diffuse streaks along 〈200〉 and 〈113〉, respectively, which we assign to rotations around the two-, three- and fourfold axes (Figs. 1 ▸ and 2 ▸). Given that only low-angle reflections are involved, we assume that observed streaks do not originate from α_1_/α_2_ radiation splitting or other experimental artefacts.

**Figure 4:**
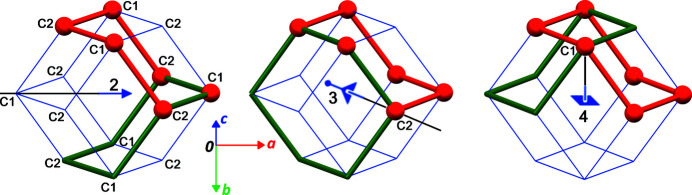
The rhombic dodecahedron (blue edges) describing the 24 disordered positions of cyclo­hexane at 255 K. On the left, atom labels are given, omitting their symmetry codes for clarity. Red and green molecules are related by twofold (left), threefold (middle) and fourfold (right) rotations. The twofold axis is viewed in the plane of projection, while the three- and fourfold axis are inclined with respect to that plane. The projection is viewed along [012], and the unit-cell origin is coincident with the centre of the polyhedron.

The simplest model based on the hypothesis of a rigid chair cyclo­hexane disordered through all crystallographic rotations includes two sites for the C atoms. Atom C1 is placed on the fourfold axis, with coordinates (*x*, 0, 0), corresponding to the Wyckoff position 24*e* (4*m.m*) in space group *Fm* 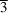 *m*, while atom C2 is placed on the threefold axis, with coordinates (*x*′, *x*′, *x*′), corresponding to the Wyckoff position 32*f* (.3*m*). By placing this asymmetric unit close to the origin, a set of 14 C atoms are connected, forming a rhombic dodecahedron, a well-studied convex polyhedron with Euler characteristic χ = 2 (Fig. 4 ▸). This polyhedron is centrosymmetric, and its centre coincides with the crystallographic inversion centre. As this polyhedron belongs to the family of edge-transitive polyhedra, all C—C bonds are equivalent and have the same bond length, as expected for cyclo­hexane. The rhombic faces, with configuration v3.4.3.4, display obtuse angles of arccos(−1/3) = ±109.47°, which accommodate *sp* ^3^-hybridized C atoms. The dihedral angle between edge-sharing rhombus is 120°, affording the expected C—C—C—C torsion angles of ±60° in cyclo­hexane.

Most importantly, the rhombic dodecahedron has full octahedral symmetry (*m* 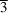 *m* or *432), and its rotation group is the chiral octahedral group 432. The chair conformation of cyclo­hexane, with symmetry 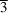 *m*, is thus compatible with the rhombic dodecahedron, and the full polyhedron is indeed generated by rotation of one chair about the elements of the rotation group 432, as reflected in the shape of the Bragg reflections, as discussed above. The molecule is then disordered over 24 positions (order of the rotation group). Symmetry-related molecules in this polyhedral cluster are depicted in Fig. 4 ▸.

Once the polyhedron describing the disorder in the plastic phase has been laid down, the structure refinement is straightforward. A single geometric parameter should actually be refined, that is the bond length C1—C2 = *d*. Since both atoms lie on special positions, only two positional parameters are used, *x* and *x*′. Using the structure factors extracted as described in the previous section, we refined an isotropic model with *SHELXL* (Sheldrick, 2015 ▸; refinement against *F* ^2^, no extinction parameter refined), including three restraints for the geometry of the polyhedron: *d* = 1.54 (1) Å, and a couple of restraints for 1,3-distances: C1⋯C1′ = 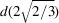 and C2⋯C2′ = 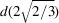 , with standard deviations of 0.03 Å, and with primed atoms generated by suitable symmetry operations. Site occupancy factors (sof) are calculated considering the Wyckoff positions and assuming that each of the 14 vertices in the polyhedron has the same probability to be occupied: sof(C1) = (24/192) × (6/14) = 3/56 and sof(C2) = (32/192) × (6/14) = 1/14. Finally, all H atoms were added in idealized positions, corresponding to special positions 96*k* (H1 bonded to C1), and 96*k* and 32*f* (H2*A* and H2*B* bonded to C2), with C—H = 0.95 Å, and with calculated displacement parameters *U*_iso_(H) = 2.8*U*_iso_(carrier C).

**Figure 5:**
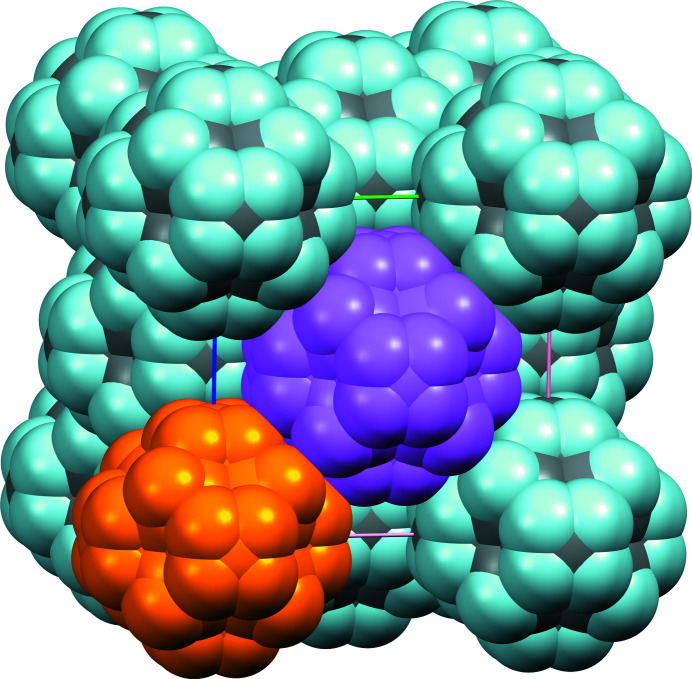
Packing structure of cyclo­hexane at 255 K, in a space-filling representation. All disordered sites for C (grey) and H (blue) atoms in one unit cell are represented with their van der Waals radii (Macrae *et al.*, 2020 ▸). Two neighbouring clusters of disordered molecules in the fcc lattice are shown in orange and magenta, with the purpose of emphasizing the contact between the two clusters. The interface separating the clusters is a ‘diagonal’ mirror plane *m* in space group *Fm* 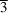 *m*.

The structure is then refined (Table 1 ▸) using five parameters and 31 independent reflections, of which ten have *F*_o_ > 4σ(*F*_o_), converging towards the expected geometry (Table 2 ▸). Notably, the refined C1—C2 bond length of 1.534 (8) Å is identical to that determined by electron diffraction, 1.535 (2) Å (Ewbank *et al.*, 1976 ▸). Displacement parameters are very high, reflecting the motions of C atoms bouncing from vertex to vertex in the polyhedral cluster. Actually, Figs. 1 ▸–3 ▸ ▸ reflect accurately the idea of Timmermans about plastic crystals: they are solids behaving like liquids over short distances (one polyhedron). From the crystallographic point of view, plastic cyclo­hexane can be seen as a liquid with long-range order, affording a diffraction pattern. The dynamic disorder being identical for every node in the lattice, the crystal structure emulates a close-packed arrangement (Fig. 5 ▸), in which the atomic sites have very low occupancies (see Table 2 ▸). As a consequence, the density is also very low, 0.85 g cm^−3^. The non-plastic phase II of cyclo­hexane has a more regular density of 1 g cm^−3^.

## Discussion and conclusions

Strangely enough, Kahn *et al.* ▸ were unable to move towards the model we propose in Table 2 ▸, probably because they did not realize that C atoms could lie on special positions. Instead, they used an asymmetric unit including three C atoms close to the origin, all in *general* positions. With such a model, the 144-vertex polyhedron describing the disorder is hugely complex, and individual cyclo­hexane molecules are hardly discernible. Actually, their polyhedron has a shape close to that of a sphere, which has Euler characteristic χ = 2, like any (convex) polyhedron whose boundary is topologically equivalent to a sphere. It is thus not surprising that they could obtain a satisfactory agreement between observed and calculated structure factors, although their structural model is far from satisfactory.

It is worth noting that the notion of ‘refinement’ for such plastic structures is of little sense, especially if least-squares methods are involved, since the data-to-parameter ratio rapidly drops to too low values. Even the identification of a suitable asymmetric unit cannot rely on mainstream approaches like direct methods, since atomic resolution is not achievable. Instead, a careful examination of data in reciprocal space, in particular the shape of the Bragg peaks, can be helpful. In 1973, this perspective was not considered by Kahn *et al.* ▸ In contrast, the 1948 ▸ article of Tutomu Oda, of limited impact because written in Japanese, is noteworthy. The abstract mentions: ‘*Besides the Bragg reflections, we observed remarkable diffuse scattering of considerable intensity, similar to that shown by cyclo­hexanol. Namely, there appear on the Laue and oscillation photographs a number of so-called diffuse spots and apparently circular diffuse haloes, which resemble to the liquid diffraction haloes*’. Nowadays, computer simulations allow the interpretation of the diffuse scattering observed in many materials. This may be achieved either in reciprocal space by considering the material as a modulated phase, or with a correlation method in direct space, using short-range chemical and atomic displacement pair-correlation parameters (Rosenkranz & Osborn, 2004 ▸; Welberry, 2022 ▸). In the case of molecular crystals, Monte Carlo and reverse Monte Carlo simulations are also a very promising approach, since they are applicable to disorder of any complexity (Welberry, 2022 ▸). However, only a few such simulations have been carried out for plastic crystals to date (for example, for α-CBr_4_; Folmer *et al.*, 2008 ▸), and the molecular dynamics associated with the disorder in these materials is not fully understood.

We also extended this study to cyclo­heptane phase I and cyclo­octane phase I (both in space group *Pm* 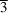 *n*). Preliminary results can be found in the Master’s thesis of the last author (Camargo, 2018 ▸; available online). We also plan to collect data at temperatures as close as possible to the melting points of these materials, and to use Cu *K*α radiation for collecting frames.

## Supplementary Material

Crystal structure: contains datablock(s) I. DOI: 10.1107/S2414314623001141/iq4001sup1.cif

Structure factors: contains datablock(s) I. DOI: 10.1107/S2414314623001141/iq4001Isup2.hkl

CheckCIF for raw data report for cyclohexane_crystal1. DOI: 10.1107/S2414314623001141/iq4001sup3.pdf

CheckCIF for raw data report for cyclohexane_crystal2. DOI: 10.1107/S2414314623001141/iq4001sup4.pdf

Stoe Stadivari data files and CBF files for cyclohexane_crystal1: https://doi.org/10.5281/zenodo.7154725

Stoe Stadivari data files and CBF files for cyclohexane_crystal2: https://doi.org/10.5281/zenodo.7155191

Metadata imgCIF file for cyclohexane_crystal1. DOI: 10.1107/S2414314623001141/iq4001img1.cif

Metadata imgCIF file for cyclohexane_crystal2. DOI: 10.1107/S2414314623001141/iq4001img2.cif

CCDC reference: 2240539

## Figures and Tables

**Table 1:** Experimental details

Raw data				
DOI	https://doi.org/10.5281/zenodo.7154725			
Data archive	Zenodo			
Data format	CBF			
				
Data collection				
Diffractometer	Stoe Stadivari			
Temperature (K)	255			
Radiation type	Ag *Kα*			
Detector type	Dectris Pilatus 100 K R			
Wavelength (Å)	0.56083			
Beam centre (mm)	41.882, 16.7			
Detector axis	−*Z*			
Detector distance (mm)	40.0			
Pixel size (mm)	0.172 × 0.172			
No. of pixels	195 × 487			
No. of scans	17			
Exposure time per frame (s)	60.0			
Swing angle (°)	Scan axis	Start angle, increment per frame (°)	Scan range (°)	No. of frames
−23.4	ω, X (χ = −55.183°, φ = −55.0°)	142.261, 1.0	47.0	47
−23.4	ω, X (χ = −30.183°, φ = −5.0°)	142.261, 1.0	47.0	47
23.4	ω, X (χ = −35.183°, φ = 150.0°)	−40.679, 1.0	77.0	77
23.4	ω, X (χ = −20.183°, φ = −85.0°)	−40.679, 1.0	77.0	77
23.4	ω, X (χ = −30.183°, φ = −105.0°)	−40.679, 1.0	77.0	77
23.4	ω, X (χ = −35.183°, φ = −35.0°)	−40.679, 1.0	77.0	77
23.4	ω, X (χ = −35.183°, φ = 75.0°)	−40.679, 1.0	77.0	77
23.4	ω, X (χ = −50.183°, φ = −160.0°)	−40.679, 1.0	77.0	77
23.4	ω, X (χ = −20.183°, φ = 25.0°)	−40.679, 1.0	77.0	77
23.4	ω, X (χ = −45.183°, φ = −55.0°)	−40.679, 1.0	77.0	77
23.4	ω, X (χ = −50.183°, φ = 15.0°)	−40.679, 1.0	77.0	77
23.4	ω, X (χ = −50.183°, φ = 125.0°)	−40.679, 1.0	77.0	77
23.4	ω, X (χ = −25.183°, φ = −110.0°)	−40.679, 1.0	77.0	77
23.4	ω, X (χ = −55.183°, φ = −170.0°)	−40.679, 1.0	67.0	67
23.4	ω, X (χ = −40.183°, φ = 140.0°)	−40.679, 1.0	67.0	67
23.4	ω, X (χ = −45.183°, φ = −150.0°)	−25.679, 1.0	32.0	32
23.4	ω, X (χ = −20.183°, φ = −100.0°)	−25.679, 1.0	32.0	32
				
Crystal data				
Chemical formula	C_6_H_12_			
*M*_r_	84.16			
Crystal system, space group	Cubic, *Fm* 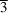 *m*			
*a* (Å)	8.712 (4)			
*V* (Å^3^)	661.1 (9)			
*Z*	4			
μ (mm^−1^)	0.03			
Crystal size (mm)	0.40 × 0.30 × 0.30			
				
Data processing				
Absorption correction	Multi-scan (*X-AREA;* Stoe & Cie, 2019 ▸)			
*T*_min_, *T*_max_	0.558, 1.000			
No. of measured, independent and observed [*I* > 2σ(*I*)] reflections	1690, 31, 10			
*R*_int_	0.018			
(sin θ/λ)_max_ (Å^−1^)	0.487			
				
Refinement				
*R*[*F* ^2^ > 2σ(*F* ^2^)], *wR*(*F* ^2^), *S*	0.080, 0.190, 1.10			
No. of reflections	31			
No. of parameters	5			
No. of restraints	3			
H-atom treatment	H-atom parameters constrained			
Δρ_max_, Δρ_min_ (e Å^−3^)	0.07, −0.16			

Computer programs: *X-AREA* (Stoe & Cie, 2019 ▸), *SHELXL2018/3* (Sheldrick, 2015 ▸) and *Mercury* (Macrae *et al.*, 2020 ▸).

**Table 2:** Refined structure of plastic cyclo­hexane at 255 K. Refined parameters are *x*, * x*′, *U*(C1) and *U*(C2)

Refined parameters	
Position of C1: (*x*, 0, 0), sof, *U*_iso_	*x* = 0.2030(18), 3/56, *U*(C1) = 0.41 (3) Å^2^
Position of C2: (*x*′, *x*′, *x*′), sof, *U*_iso_	*x*′ = 0.1019 (10), 1/14, *U*(C2) = 0.35 (3) Å^2^
	
Cyclo­hexane geometry	
C—C bond length	1.534 (8) Å
C—C—C bond angles	109.9 (17), 109.2 (8)°
C—C—C—C torsion angles	−59.7 (10), 59.7 (10)°
