# Supplementary material for: Cyclo­hexane plastic phase I: single-crystal diffraction images and new structural model
Source: IUCrdata. 2023 Mar 2;8(Pt 3):x230114. doi: 10.1107/S2414314623001141 (PMC10171323; doi:10.1107/S2414314623001141)
Supplement: Supplementary file 3 [file x-08-x230114-sup3.pdf]

checkImgCIF report

Powered by https://github.com/jamesrhester/ImgCIFHandler.jl

```
 ImgCIF checker version 2022-08-04

Running checks (no image download)
========================================


Testing: Required items: PASS

Testing: Data source: PASS

Testing: Axes defined: PASS

Testing: Our limitations: PASS

Testing: Detector translation: PASS

Testing: Scan range: PASS
   Range/increment match number of frames 33.0 for scan SCAN01 (expected 33.0)
   Range/increment match number of frames 29.0 for scan SCAN02 (expected 29.0)
   Range/increment match number of frames 29.0 for scan SCAN03 (expected 29.0)
   Range/increment match number of frames 27.0 for scan SCAN04 (expected 27.0)
   Range/increment match number of frames 20.0 for scan SCAN05 (expected 20.0)
   Range/increment match number of frames 19.0 for scan SCAN06 (expected 19.0)
   Range/increment match number of frames 14.0 for scan SCAN07 (expected 14.0)
   Range/increment match number of frames 12.0 for scan SCAN08 (expected 12.0)

Testing: All frames present: PASS
   All frames present and correct for SCAN01
   All frames present and correct for SCAN02
   All frames present and correct for SCAN03
   All frames present and correct for SCAN04
   All frames present and correct for SCAN05
   All frames present and correct for SCAN06
   All frames present and correct for SCAN07
   All frames present and correct for SCAN08

Testing: Detector surface axes used properly: PASS

Testing: Pixel size and origin described correctly: PASS

Testing: Check calculated beam centre: FAIL
   Unable to carry out test, assume missing or bad value

Testing: Check principal axis is aligned with X: PASS

Testing: All archives are accessible: PASS

Running checks with downloaded images
========================================
```
