# Supplementary material for: Cyclo­hexane plastic phase I: single-crystal diffraction images and new structural model
Source: IUCrdata. 2023 Mar 2;8(Pt 3):x230114. doi: 10.1107/S2414314623001141 (PMC10171323; doi:10.1107/S2414314623001141)
Supplement: Supplementary file 4 [file x-08-x230114-sup4.pdf]

checkImgCIF report

Powered by https://github.com/jamesrhester/ImgCIFHandler.jl

```
 ImgCIF checker version 2022-08-04

Running checks (no image download)
========================================


Testing: Required items: PASS

Testing: Data source: PASS

Testing: Axes defined: PASS

Testing: Our limitations: PASS

Testing: Detector translation: PASS

Testing: Scan range: PASS
   Range/increment match number of frames 47.0 for scan SCAN01 (expected 47.0)
   Range/increment match number of frames 47.0 for scan SCAN02 (expected 47.0)
   Range/increment match number of frames 77.0 for scan SCAN03 (expected 77.0)
   Range/increment match number of frames 77.0 for scan SCAN04 (expected 77.0)
   Range/increment match number of frames 77.0 for scan SCAN05 (expected 77.0)
   Range/increment match number of frames 77.0 for scan SCAN06 (expected 77.0)
   Range/increment match number of frames 77.0 for scan SCAN07 (expected 77.0)
   Range/increment match number of frames 77.0 for scan SCAN08 (expected 77.0)
   Range/increment match number of frames 77.0 for scan SCAN09 (expected 77.0)
   Range/increment match number of frames 77.0 for scan SCAN10 (expected 77.0)
   Range/increment match number of frames 77.0 for scan SCAN11 (expected 77.0)
   Range/increment match number of frames 77.0 for scan SCAN12 (expected 77.0)
   Range/increment match number of frames 77.0 for scan SCAN13 (expected 77.0)
   Range/increment match number of frames 67.0 for scan SCAN14 (expected 67.0)
   Range/increment match number of frames 67.0 for scan SCAN15 (expected 67.0)
   Range/increment match number of frames 32.0 for scan SCAN16 (expected 32.0)
   Range/increment match number of frames 32.0 for scan SCAN17 (expected 32.0)

Testing: All frames present: PASS
   All frames present and correct for SCAN01
   All frames present and correct for SCAN02
   All frames present and correct for SCAN03
   All frames present and correct for SCAN04
   All frames present and correct for SCAN05
   All frames present and correct for SCAN06
   All frames present and correct for SCAN07
   All frames present and correct for SCAN08
   All frames present and correct for SCAN09
   All frames present and correct for SCAN10
   All frames present and correct for SCAN11
   All frames present and correct for SCAN12
   All frames present and correct for SCAN13
   All frames present and correct for SCAN14
   All frames present and correct for SCAN15
   All frames present and correct for SCAN16
   All frames present and correct for SCAN17

Testing: Detector surface axes used properly: PASS

Testing: Pixel size and origin described correctly: PASS

Testing: Check calculated beam centre: FAIL
   Unable to carry out test, assume missing or bad value
```

```
Testing: Check principal axis is aligned with X: PASS

Testing: All archives are accessible: PASS

Running checks with downloaded images
========================================
```
